# Recent Studies in Albuminuria*Read before the Buffalo Medical Club, April 25th, 1880.

**Published:** 1880-09

**Authors:** H. S. Kilbourne

**Affiliations:** Asst. Surgeon U. S. Army


### RECENT STUDIES IN ALBUMINURIA.*

BY H. S. KILBOURNE, M. D., ASST. SURGEON U. S. ARMY.

The views of the majority of the profession concerning the diagnostic value of albuminuria might be stated in the words of Dr. Saundby, as follows: (*British Medical Journal*, May 10th, 1879.) The presence of albumen in the urine is generally indicative of renal disease, especially where it is persistent and is not the accompaniment of more prominent pathological conditions, such as fever, severe inflammatory disease, or affections of the circulatory system.

Dr. Saundby examined the urine of one hundred and forty-six male patients, taken seriatim, as they presented themselves for treatment in the out-patient department of the General Hospital, Birmingham. Of this number, the urine of 105 contained albumen in "more or less" quantity. Of 105 cases, 66 were tabulated as granular kidney, and one as fatty kidney. "This number," he says, "seems large, but the diagnosis was carefully made and the proportion does not much exceed the post-mortem room statistics of the hospital."

*Read before the Buffalo Medical Club, April 25th, 1880.

The (105) patients were of all ages, from 10 to 80 years. Excluding the (66) cases of organic kidney disease, the remainder (39) are tabulated under 18 other headings; dyspepsia (8 cases); debility (7 cases); phthisis (7 cases); morbus cordis (6 cases); syphilis, bronchitis, tonsillitis, acne, (of each 2 cases); epilepsy, laryngitis and other acute and chronic diseases, (one case each.)

Dr. Saundby tested these urines on each appearance of the patients, and proved (*sic*) its persistence for weeks and months. The microscopical examinations revealed, usually, nothing. In one or two cases there were oxalates, and in one or two, a few hyaline casts, which he regarded as of no pathological significance.

From additional details given, it appears that these investigations were conducted with care and painstaking, but certain particulars are wanting, and the results so loosely stated as to impair their value. What, for instance, is to be inferred from the statement that the proportion (of kidney disease) 66 in a total of 350 men, *women* and *children*, does not much exceed the post-mortem room statistics of the hospital, after the preliminary statement of the analysis and tabulation of results in male patients only? And are we to infer that in 66 cases of organic disease of the kidney, the urines revealed nothing under the microscope?

In connection with these investigations the *Journal* (*idem*) refers to the experiments of Dr. Marcacci, reported to the Medical Society of Florence, in which he proves by a series of observations on himself, that albumen is present in physiological urine under certain conditions of diet and exercise.

The more extended and accurate observations of Leube of Erlangen, are summarized as follows: Leube undertook his researches on the garrison of Erlangen and took the necessary precaution to exclude cases of blennorrhagia. Fresh urine was filtered and a certain quantity boiled; the remainder was treated with nitric acid, both being compared with the intact urine on a block tablet. To the urine which showed opacity, a small quantity of acetic acid was added to precipitate the deposit.

The precipitate was washed and treated with Millou's fluid and also tested with Liq. Potass. Leube examined the night urine of 119 soldiers. The number of observations was 154, which were thus divided: 90 soldiers were examined once, 23 were examined twice on two different days, and 6 were examined three times at intervals of three days. Of 154 examples of nocturnal urine, only a small quantity was found in 5 cases and in only one case in notable proportion.

Examination of the urine secreted during the day, after military exercise, and in the months of June, July and August gave different results. Of 5 soldiers who had shown albumen in the night urine, a larger quantity was found during the day, and albumen was found in 18 soldiers who had not shown any in the night urine. Stated as a percentage, the morning urine was albuminous in 5 of 119 soldiers, i. e., in 4.2 per cent. That of the middle of the day was albuminous in 19 of 119, i. e., 16 per cent. The day urine was only albuminous in 14 of 19, i. e. 11.8 per cent., and, finally, the urine was equally albuminous in 5 of 119, i. e., 4.2 per cent.

The quantity of albumen in the urine most heavily loaded was 38 milligrammes per cent.

Such are the facts which seem to prove the possibility of albuminuria in the physiological state.

At the International Medical Congress, held in Amsterdam, September, 1879, Prof. Semmola in a *resume* (*British Medical Journal*, September 27, 1879) of his researches in 300 cases of Bright's disease, presented to the medical section, said: The passage of albumen into the urine may take place through the three physiological factors that preside over the renal functions, viz: *a*, chemical constitution of the blood; *b*, degree of pressure; *c*, condition of the histological elements of the kidney. Accordingly there are three classes of albuminuria, viz: *a*, dyscrasic albuminuria; *b*, mechanical albuminuria; *c*, mechanical albuminuria produced by irritation, i. e., by histological changes in the kidney.

Bright's disease cannot be placed exclusively under either of these heads. It is a mixed albuminuria. Its complicated etiological mechanism contains all the three mechanisms of the other classes of albuminuria, and it forms a pathological specialty that has nothing whatever to do with the other classes of this affection. The most characteristic phenomenon of Bright's disease, he thinks, is the defective formation of urea. This would be indicated, not by the specific gravity of the urine, but by the weight of urea excreted.

The frequency of albuminuria in adolescents has been observed by Dr. Clement Dukes, Physician to Rugby school, who reports 10 cases (*British Medical Journal*, Nov. 30, 1878.) In three of the cases there was the history of an antecedent scarlatina. In the majority of cases the albumen appeared transiently, in others it was intermittent in appearance and in a few instances it appeared persistently for several months. In one case the quantity was "one-third," in another "one-fourth." The appearance of albumen was accompanied by headache, fever, a coated tongue, malaise, and, in some cases, syncope. All the cases recovered, or were lost sight of, by removal from the school.

Dr. George Johnson, in commenting on Dr. Duke's cases, says (*British Medical Journal*, Dec. 13, 1879) that a large proportion of such cases may be traced to some probable exciting cause and that the presence of even a trace of albumen in the urine is always pathological and never physiological.

Dr. Peabody reports a case (*N. Y. Medical Record*, April 17, 1880) in which the patient was suffering from an injury of the head, from which he shortly after died. The urine during life was found loaded with albumen, but, a careful examination of the kidneys, *post-mortem*, showed them to be absolutely healthy.

Dr. Brunton, in an interesting article on the use of arsenic in albuminuria, says (*Braitwait's Retrospect*, Jan., 1878): True albuminuria consists in the passage of serum albumen, which is a normal constituent of the blood into the urine. It depends either upon alterations in the structure of the kidney, or interference with the circulation in it, or on both. It has been supposed that great increase in the pressure of the blood within the renal glomeruli will cause albumen to appear in the urine, but the experiments of Stockvis appear to disprove this supposition. He found that no increase in the arterial pressure, either generally throughout the body, or in the kidney alone, would produce it. He raised the general pressure by compressing the aorta and other large arteries and he raised the pressure in the kidney itself by dividing the vaso-motor nerves of the organ, so that the renal arteries dilated and allowed much more blood than usual to pour into the kidneys. In neither case did he find any albuminuria, but the result was very different when he interfered with the venous circulation of the kidney.

An obstruction to the return of the blood through the renal veins was sufficient to cause albuminuria. It came on when the renal veins were tied, when the vena cava was plugged, or when the movements of the heart were interfered with by a ball passed into the right ventricle, or when quantities of fluid were quickly injected into the jugular vein. Venous congestion, then, is the cause of albuminuria, depending on alterations in the circulation.

The second cause of true albuminuria is alteration in the structure of the kidney, and these alterations may affect the vessels and tubules, or the connective tissue stroma in which they are imbedded.

In the waxy kidney, the vessels are affected and the structure of their walls is changed.

It seems now improbable that the altered structure of the vessels may permit the serum-albumen to transude through them in the same way, as the vessels in their normal condition permit the transudation of egg-albumen. In desquamative nephritis we may suppose that the albumen finds its way into the uriniferous tubules because the epithelium has been removed. Even in cases where albuminuria depends on organic disease the quantity of albumen varies with the state of the circulation.

Dr. Mahomed's conclusions are summarized (*American Journal Medical Sciences*, July, 1879) in the following propositions:

1. Albuminuria, though occasionally produced by other causes, is generally the result of increased pressure in the capillaries of the kidneys, either venous or arterial.

2. Neither albuminuria nor dropsy are usually present in chronic Bright's disease; when present they indicate acute or epithelial changes.

In order to arrive at the practical value of the foregoing investigations, as regards the diagnostic value of albuminuria, it may be noted that in the so-called physiological albuminuria the *quantity* of albumen is commonly small. That in the albuminuria of adolescents in but few cases was there albumen in quantity that was persistent. That in Dr. Saundby's cases the quantity of albumen is not given with precision, and generally as the result of these observation that albuminuria is associated with a variety of diseases, and that it may occur where no other evidence of disease is known to exist, and that (according to Mahomed) albumen may be absent from the urine in organic disease of the kidney.

In so far as the conclusions of these observations lessen the value of albuminuria as evidence of renal disease, they are to be compared with the careful and convincing observations of Parkes (quoted by Roberts) and with the extensive clinical experience of Roberts himself, Dr. G. Johnson and others.

It should not be forgotten that albuminuria is but one of the clinical features of organic disease of the kidney or that only under certain conditions does it become of preponderating value in the diagnosis of diseases of this class.

In the present state of our knowledge it would appear that we cannot agree with Dr. Saundby in the statement that albuminuria is a phenomenon which may be present under such a variety of conditions that, *per se*, it affords no indications of renal disease. A safer rule of practice would be, (excluding cases that of error arising from disease outside the kidneys) albumen in large quantity and persistent in occurrence, is seldom found in the urine except in Bright's disease.

